# Innovations in ultrasound training in obstetrics

**DOI:** 10.1007/s00404-024-07777-8

**Published:** 2024-10-15

**Authors:** Agnes Wittek, Brigitte Strizek, Florian Recker

**Affiliations:** Department of Obstetrics and Prenatal Medicine, University Hospital Bonn, Venusberg Campus 1, 53127 Bonn, Germany

**Keywords:** Education, Ultrasound training, Obstetrics, Ultrasound

## Abstract

**Introduction:**

Ultrasound technology is critical in obstetrics, enabling detailed examination of the fetus and maternal anatomy. However, increasing complexity demands specialised training to maximise its potential. This study explores innovative approaches to ultrasound training in obstetrics, focussing on enhancing diagnostic skills and patient safety.

**Methods:**

This review examines recent innovations in ultrasound training, including competency-based medical education (CBME), simulation technologies, technology-based resources, artificial intelligence (AI), and online-learning platforms. Traditional training methods such as theoretical learning, practical experience, and peer learning are also discussed to provide a comprehensive view of current practises.

**Results:**

Innovations in ultrasound training include the use of high-fidelity simulators, virtual reality (VR), augmented reality (AR), and hybrid-learning platforms. Simulation technologies offer reproducibility, risk-free learning, diverse scenarios, and immediate feedback. AI and machine learning facilitate personalised-learning paths, real-time feedback, and automated-image analysis. Online-learning platforms and e-learning methods provide flexible, accessible, and cost-effective education. Gamification enhances learning motivation and engagement through educational games and virtual competitions.

**Discussion:**

The integration of innovative technologies in ultrasound training significantly improves diagnostic skills, learner confidence, and patient safety. However, challenges such as high costs, the need for comprehensive instructor training, and integration into existing programs must be addressed. Standardisation and certification ensure high-quality and consistent training. Future developments in AI, VR, and 3D printing promise further advancements in ultrasound education.

**Conclusion:**

Innovations in ultrasound training in obstetrics offer significant improvements in medical education and patient care. The successful implementation and continuous development of these technologies are crucial to meet the growing demands of modern obstetrics.

## What does this study add to the clinical work

**Table:** 

This study explores innovative approaches to ultrasound training in obstetrics, integrating technologies like high-fidelity simulators, virtual reality, augmented reality, and artificial intelligence. These advancements improve diagnostic accuracy, learner confidence, and patient safety while providing flexible, personalized learning experiences. The study also emphasizes the need for standardization and certification to ensure consistent, high-quality training across medical professionals. These innovations are crucial in addressing the growing complexity of modern ultrasound technology.	

## Introduction

In recent decades, ultrasound technology has become an indispensable tool in obstetrics [1–3]. It enables detailed examination of the foetus and maternal anatomy, which helps to improve prenatal care and the early detection of complications. However, with the increasing complexity of ultrasound technology, the need for specialised training is also increasing.

Ultrasound (US) is not only a tool in the diagnostic process but also a powerful instrument for patient management and treatment monitoring. The impact of US ranges from prenatal screening and the detection of foetal anomalies to the diagnosis of complex gynaecological disorders, continuously reshaping the field of gynaecology and improving patient care.

However, realising the full potential of US in obstetric–gynaecological (OG/GYN) practice goes beyond the technological capabilities of the equipment. Deciphering the fine detail and complexity contained in US images requires a level of expertise and precision that can only be achieved through structured, comprehensive and immersive training. Despite this clear need, the optimal strategy for implementing OB/GYN US education remains a complex problem influenced by many factors. It is therefore critical that we strive for an approach that is flexible, inclusive, and adaptive [4].

The introduction of ultrasound technology in obstetrics began in the 1950s. Originally, the technology was primarily used to determine foetal size and position. In the decades that followed, the equipment was continually refined so that today high-resolution 2D, 3D, and 4D images are available. These technological advances have required training programmes to be adapted accordingly. These should be based on the following points:

- Competency-based medical education (CBME): focus on mastery of essential skills, individualised learning paths
- Simulations: Risk-free practical experience, preparation for clinical assignments
- Technology-based resources: e-modules, mobile applications, flexible learning opportunities
- Artificial intelligence (AI): immediate feedback, simulation-based learning environments, improvement of diagnostic precision
- Regular feedback and accreditation: ensuring long-term quality, saving time through automation
- High standards: Consistent and outstanding OB/GYN ultrasound care and safety
- Adaptation to innovations: Continuous further development and adaptation to new technologies

This article examines the latest innovations in ultrasound training in obstetrics, which aim to improve the diagnostic skills of medical professionals whilst enhancing patient safety.

## Traditional methods of ultrasound training

Ultrasound training in obstetrics has evolved over decades and is based on proven traditional methods. These methods can be divided into several key areas: theoretical training, practical experience under supervision and peer learning [2].

Theoretical training forms the foundation of every ultrasound-training programme. It includes lectures, seminars and textbooks that impart basic knowledge about the physics of ultrasound, the operation of the equipment and the interpretation of ultrasound images. The theoretical part of the training also includes knowledge of the anatomy and pathophysiology of the female pelvis and foetus. This sound knowledge base enables students to understand and apply the principles and techniques of ultrasound [5].

Lectures and seminars are often given by experienced medical specialists and scientists who share their expertise and clinical experience. Textbooks and scientific articles complement the lectures by providing the detailed information and visual material needed to understand complex concepts.

Practical experience is an essential part of ultrasound training. This often begins with observing experienced professionals during ultrasound examinations. Learners observe how the experts handle the equipment, perform the examinations and interpret the images. This observation phase is crucial as it gives learners the opportunity to see and understand the practical aspects of ultrasound before they become active themselves.

After the observation phase, students begin to carry out their own examinations under direct supervision. This is done in a clinical environment where they receive real-time feedback and instructions from the experienced professionals. This supervision is essential to ensure that learners are applying the correct techniques whilst ensuring the safety and well-being of patients.

Peer learning, i.e. learning from and with colleagues, also plays an important role in traditional ultrasound training. In many training programmes, students are encouraged to work in groups and support each other in performing ultrasound examinations [6]. This can be done through joint-case discussions, practice sessions, and peer assessment.

Peer learning allows learners to benefit from each other by observing and discussing different approaches and techniques. It also promotes teamwork and communication, which are essential in clinical practice. In addition, new insights can often be gained and uncertainties clarified through the exchange with colleagues [7].

With the increasing complexity of ultrasound technology and the growing number of pregnant women requiring high-quality antenatal care, there is an urgent need for innovative training approaches. These approaches should ensure that medical professionals are fully trained and able to use the technology effectively and safely.

## Simulation technologies in ultrasound training

The use of simulation technologies in ultrasound training has grown significantly in recent years. These technologies provide a safe, controlled and reproducible environment in which learners can develop and refine their skills without the risk of harming patients [8]. Simulation technologies can be divided into different categories, including highly realistic simulators, virtual reality (VR), augmented reality (AR), and hybrid-learning platforms.

*Highly realistic (high-fidelity) simulators*

Highly realistic ultrasound simulators are specially developed devices that reproduce the anatomy and physiology of the human body in great detail [9]. These simulators use realistic ultrasound images and allow learners to practice a variety of scenarios, from normal to pathological findings [10]. Examples of such simulators are “SonoSim”, “Medaphor ScanTrainer” and “CAE VIMEDIX”.

These simulators offer several decisive advantages:

- Reproducibility: Learners can practise identical scenarios repeatedly to improve their skills [11].
- Risk-free learning: Mistakes can be made and corrected without any risk to real patients.
- A wide range of scenarios: From normal pregnancy to rare complications, various clinical situations can be simulated.
- Direct feedback: Many simulators offer immediate feedback and detailed performance evaluations, which speeds up the learning process considerably.

*Virtual reality (VR)*

Virtual reality is a technology that creates immersive, three-dimensional environments in which learners can interact. In ultrasound training, VR enables a deeper and more realistic experience by simulating the physical and visual aspects of the ultrasound examination [12, 13]. The following applications exist with corresponding benefits:

- Interactive learning modules: VR-based learning modules can represent complex anatomical structures and pathological conditions in a way that is difficult to replicate in the real world [14].
- Collaborative training: VR allows multiple learners to train simultaneously in a virtual environment, which promotes teamwork and collaborative decision-making.
- Reduction of the learning curve: Studies have shown that VR-based training can shorten the learning curve as it offers a more intensive and focussed learning environment.

*Augmented reality (AR)*

Augmented reality supplements the real world with digital information and overlays. In ultrasound training, AR can be used to provide real ultrasound examinations with additional information that makes it easier to understand and interpret the images [15]. The following advantages are offered by AR applications in the field of ultrasound training [16]:

- Live overlays: AR devices such as the “Microsoft HoloLens” can display additional information during a real ultrasound examination, such as anatomical landmarks or pathological findings superimposed on the ultrasound image.
- Interactive tutorials: AR-based tutorials can provide step-by-step instructions that are displayed directly in the learner’s field of vision, making the learning process more intuitive and accessible.
- Extended diagnostic options: By combining real and virtual data, learners can better understand and comprehend complex diagnoses.

## Hybrid-learning platforms

Hybrid-learning platforms combine different technologies and methods to create a comprehensive and flexible learning environment [17]. These platforms often integrate simulators, VR, AR, and traditional e-learning modules.

- Holistic approach: Hybrid platforms offer a combination of theoretical knowledge and practical skills, reinforced by the use of simulation and virtual technologies.
- Personalised learning paths: Learners can track their own progress and create personalised training plans based on their individual strengths and weaknesses.
- Continuous feedback: By integrating various technologies, learners receive continuous and differentiated feedback that supports and improves their learning process.

## 3D printing in US education

3D printing has had a significant impact on various medical disciplines in recent years [18]and ultrasound training in obstetrics is also increasingly benefiting from this technology. 3D printing enables the creation of physical models that can serve as realistic training aids to improve the skills of medical students and professionals. One of the most basic and valuable uses of 3D printing in ultrasound training is the production of anatomical models [19]. These models can include detailed replicas of the female pelvic anatomy, the foetus at various stages of development and specific pathological conditions. Such models provide learners with the opportunity to haptically grasp and better understand the complex anatomy.

**Fig. 1:**
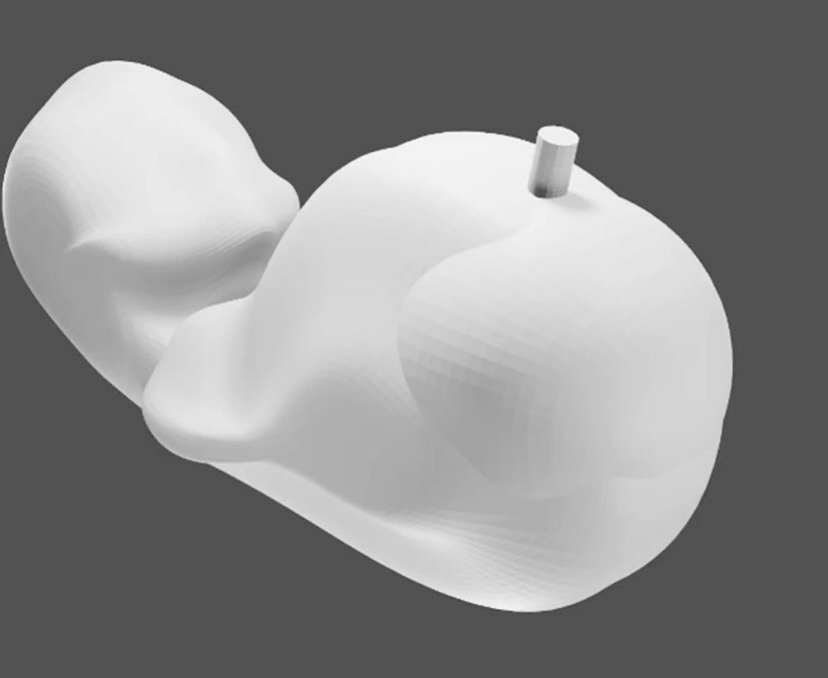
3D-printed modelling of a foetus with LUTO disease using modern rendering with FormLabs™

- Realistic representation: 3D-printed models provide a highly realistic representation of anatomical structures, which improves learners’ understanding and visual imagination (Fig. 1). One example is a 3D-printed model of the foetal heart that learners can use to study the heart structure and possible anomalies.
- Interactive learning opportunities: These models can be used in combination with ultrasound equipment to improve hand–eye coordination and image interpretation skills. Learners can place the transducers on the models and compare the images produced with the physical structures.
- Individualised training: Models can be created based on real patient data to replicate specific cases and pathologies. This enables personalised training that focuses on the specific needs and interests of learners.

The future of 3D printing in ultrasound training is promising. As technology advances and experience grows, models will become more realistic and versatile. Combined with other innovative technologies such as virtual reality and artificial intelligence, 3D-printed models could become part of comprehensive training programmes that offer a deep and interactive learning experience [20]. An exciting future scenario is the development of ‘intelligent’ models that are equipped with sensors and can provide feedback in real time. These models could simulate the physical and biological reactions of the human body and offer learners an even more realistic training experience.

Research in the field of simulation technologies for ultrasound training is active and dynamic. New developments aim to further improve the accuracy and realism of simulations whilst increasing accessibility and ease of use. Numerous studies have shown that simulation training significantly improves diagnostic skills and learner confidence [21]. Comparisons between traditional training and simulation-based training often show that the latter group has a faster and deeper learning experience. Likewise, long-term studies on the effectiveness of simulation training have shown that the acquired skills are retained over time and can be applied in clinical practice [11].

Despite the numerous advantages, simulation technologies face a number of challenges. The main challenges include the high cost of purchasing and maintaining the equipment, the need for comprehensive training of instructors and the integration of these technologies into existing training programmes.

The purchase of high-end simulators and VR/AR devices is expensive. Educational institutions therefore need to carefully consider how they can integrate these technologies into their budgets. Long-term investments in simulation technologies must be supported by a detailed cost–benefit analysis.

To maximise the benefits of simulation technologies, trainers must also receive comprehensive training. This includes both the technical handling of the devices and the ability to give effective feedback and support learners [22].

The integration of new technologies into existing training programmes requires careful planning and implementation. It is important that the new methods are seamlessly combined with traditional approaches to ensure comprehensive and coherent training.

Simulation technologies offer enormous benefits for ultrasound training in obstetrics. By utilising highly realistic simulators, virtual and augmented reality and hybrid-learning platforms, learners can develop and refine their skills in a safe and controlled environment. Despite the challenges associated with cost and integration, these technologies offer the potential to significantly improve the quality of ultrasound education and increase diagnostic skills and patient safety [8]. The future of ultrasound education will depend heavily on the successful implementation and further development of these innovative technologies.

## Online-learning platforms and e-learning

Digitalisation has fundamentally changed the educational landscape and also offers immense opportunities in ultrasound training in obstetrics. Online-learning platforms and e-learning methods make it possible to disseminate knowledge in a flexible, accessible and often more cost-effective way. These modern-learning approaches include interactive online courses, webinars, virtual classrooms, mobile-learning applications and social-learning networks [23].

Interactive online courses are at the heart of e-learning. These courses combine multimedia resources such as videos, animations, interactive modules and quizzes to support the learning process. Platforms such as Coursera, Udemy and specialised medical-learning platforms offer ultrasound-training courses developed by renowned universities and experts. These courses are designed to provide comprehensive training that covers both theoretical and practical aspects.

The flexibility of these courses is one of their biggest advantages. Learners can complete the courses at their own pace and have round-the-clock access to the materials. This is particularly beneficial for medical professionals with irregular working hours who cannot stick to fixed course times. The broad coverage of topics ensures that both beginners and advanced users can find the content relevant to them. Interactive elements such as virtual ultrasound examinations and instant feedback allow learners to apply their knowledge practically and check their progress themselves.

An example of such a course is the “Introduction to Obstetric Ultrasound” on Coursera, which was developed by a leading medical school and offers a combination of video lectures, quizzes and practical exercises. This course covers the basic principles of ultrasound, the anatomy of the female pelvis and fetus, and specific techniques for detecting abnormalities.

Webinars and online seminars provide a platform for live interaction between trainers and learners. These formats allow experts to share their knowledge globally and discuss current developments and research findings. During the COVID-19 pandemic, platforms such as Zoom and Microsoft Teams have proven to be indispensable tools that have made it possible to maintain educational operations.

A significant advantage of webinars is the opportunity to interact directly with the trainers, ask questions and receive immediate answers. This promotes a deeper understanding of the subject matter and allows learners to clarify specific issues and uncertainties. Many webinars also offer interactive components such as live demonstrations of ultrasound examinations and case discussions in which participants are actively involved.

For example, the International Society of Ultrasound in Obstetrics and Gynaecology (ISUOG) regularly organises webinars on various topics, including advanced foetal echocardiography techniques and prenatal diagnosis of rare anomalies [24]. These webinars are often available free of charge or for a small fee and provide participants with access to leading experts in their field.

Virtual classrooms combine the benefits of online courses and webinars by providing a structured learning environment facilitated by experienced instructors. These classrooms allow learners to participate in live sessions where lectures are given, discussions are held and practical exercises are carried out.

Hybrid learning models, which include both online and face-to-face components, offer a comprehensive training experience. These models allow learners to learn the theoretical fundamentals online and then practice practical skills in face-to-face sessions. This can be particularly beneficial in ultrasound training, as learners can combine the flexibility of online learning with the necessary hands-on experience.

One example of a hybrid learning model is the “Ultrasound in Obstetrics and Gynecology” programme of the American Institute of Ultrasound in Medicine (AIUM)[25]. This programme offers a series of online modules that cover the theoretical basics, followed by practical workshops in which participants perform ultrasound examinations under the guidance of experienced professionals.

Mobile-learning applications offer a flexible way to access learning content anytime, anywhere. These apps can include interactive learning modules, quizzes, case studies and even simulations. They are especially useful for medical professionals who want to continue their education on the go. An example of a mobile-learning application is “SonoSim”, which offers a wide range of ultrasound learning modules [26]. This app contains realistic simulations and interactive learning materials to help learners develop and refine their skills. The ability to access these resources at any time makes it easier to continuously learn and progress.

Social-learning networks and online communities provide a platform for the exchange of knowledge and experience. Platforms such as Facebook groups, LinkedIn and specialised forums allow learners to ask questions, discuss case studies, and exchange ideas with other professionals.

These networks promote peer learning and offer a valuable support community. By sharing experiences and best practises, learners can benefit from the insights and knowledge of their colleagues. One example of such a community is the Facebook group “Obstetric Ultrasound Enthusiasts”, where professionals from around the world discuss cases, ask questions and share valuable resources.

Despite the numerous advantages, online learning platforms and e-learning in ultrasound training face a number of challenges. One of the biggest challenges is the quality assurance of the courses offered. It is important to ensure that the learning content is up-to-date, scientifically sound and taught by qualified professionals.

Another problem is the technical infrastructure. Not all learners have access to fast internet connexions or the necessary devices to participate in online courses and webinars. This can affect accessibility and equal opportunities. However, future developments could address these challenges. Advances in internet technology and the proliferation of mobile devices are likely to improve accessibility. In addition, accreditation and certification programmes could help to ensure the quality of online-learning opportunities.

The integration of AI and adaptive learning systems could also further increase the efficiency and effectiveness of e-learning. These systems could create personalised-learning paths that are tailored to the individual progress and needs of learners, thereby optimising the learning process.

## Gamification in ultrasound training

Gamification, the application of game-like elements in a non-game context, has proven to be an effective method for increasing learning motivation and improving learning. In ultrasound training in obstetrics, gamification can take various forms, from educational games and quiz apps to simulation-based educational games and virtual competitions [27]. This section explores the different approaches to gamification, their benefits and challenges, and the future prospects of these innovative learning methods.

Educational games and quiz apps are one of the simplest and most popular forms of gamification. They offer a fun way to test and deepen knowledge by challenging learners to answer questions and solve tasks that test their knowledge and skills. An example of a successful educational game app is “SonoGames”, an application specifically designed for medical students and professionals to test and expand their knowledge of ultrasound in obstetrics [28]. The app contains a variety of quiz questions covering different aspects of ultrasound diagnostics, from the anatomy of the female pelvis to the detection of foetal anomalies. By solving these quizzes, learners can consolidate their knowledge and absorb new information at the same time.

The advantages of educational games and quiz apps lie in their ability to turn learning into an entertaining and engaging activity. They encourage active engagement with the learning material and improve retention of knowledge through repeated practice. They also offer learners the opportunity to track their progress and work specifically on their weaknesses.

Simulation-based learning games.

Simulation-based learning games go one step further by recreating realistic scenarios and clinical situations where learners can practice their skills in a safe and controlled environment. These games combine elements of simulation with gamified challenges to create a deep and immersive learning experience. A notable example of a simulation-based learning game is The Simulab’s TraumaMan, a training system used not only in obstetrics but also in surgical training. Although it was originally developed for the training of surgical emergency techniques, the concept can be transferred to ultrasound training in obstetrics. In such a game, learners could play through various antenatal scenarios, from routine examinations to emergency situations, sharpening their diagnostic and decision-making skills.

The benefits of simulation-based learning games lie in their ability to place learners in realistic clinical environments where they can practice and hone their skills. They provide a risk-free environment where mistakes can be made and learnt from without putting patients at risk. They also encourage learners’ critical thinking and problem-solving skills by confronting them with complex and unexpected situations. Virtual competitions and leaderboards are other gamified approaches that can energise the learning process by introducing elements of competition. These methods capitalise on people’s natural need for recognition and success to increase learner motivation and engagement.

One example of the use of competitions in ultrasound training is the “SonoChallenge”, a virtual competition in which medical students and professionals compete against each other in various ultrasound examinations. The participants have to make accurate diagnoses and demonstrate their skills within a limited time frame. The best performers are ranked and receive awards or certificates recognising their achievements.

The benefits of virtual competitions lie in their ability to push learners to perform at their best and make the learning process more dynamic and exciting. They encourage continuous learning and skill improvement as learners strive to improve their rankings. They also provide a platform for sharing knowledge and experiences amongst participants, which can lead to a stronger community and collaboration.

Despite the numerous advantages, gamified learning approaches face a number of challenges. One of the biggest challenges is developing games and apps that are both entertaining and educationally valuable. It is important that the games are not only fun but also deliver relevant and accurate learning content.

Another problem is the acceptance of gamification methods by learners and trainers. Some medical professionals may be sceptical about the use of games in training and question the effectiveness of these methods. It is therefore crucial that the benefits of gamification are supported by scientific studies and empirical data [29].

To overcome these challenges, it is important that the development of gamified learning platforms is supported by experts from various fields, including medicine, education, and game development. This interdisciplinary collaboration can ensure that the games are both didactically valuable and of high technical quality. In addition, regular evaluations and feedback loops should be built in to continuously improve the games and adapt them to the needs of the learners.

The future of gamification in ultrasound training is promising. As technology advances, particularly in the areas of AI and virtual reality, the possibilities for immersive and interactive learning games will continue to grow. Future developments could include adaptive learning games that adjust to individual learning progress and offer personalised challenges.

In addition, the integration of social networks and online communities into gamified learning platforms could further promote collaboration and knowledge sharing amongst learners. The use of AI could also provide detailed analyses of learning behaviour and performance to enable targeted improvements and individual support.

## Artificial intelligence (AI) and machine learning in ultrasound training

AI and machine learning (ML) have the potential to revolutionise ultrasound training in obstetrics [30]. These technologies offer new opportunities to improve training by enabling personalised-learning pathways, automated-image analysis and instant feedback [31]. In this section, the various applications of AI and ML in ultrasound training are described in detail and their benefits and challenges are discussed.

One of the most promising applications of AI in ultrasound training is automated-image analysis. AI systems can analyse ultrasound images in real time and provide diagnostic information. This can be particularly useful for students who are still learning the intricacies of image interpretation [32]. For example, ML algorithms can be used to segment and label different structures in the ultrasound image, helping learners to identify the relevant anatomical structures more quickly and accurately. In addition, AI systems can be trained to recognise abnormal findings and alert learners to potential problems, such as foetal anomalies like heart defects or spina bifida. The use of such technologies offers several key benefits, including high accuracy and consistency in image analysis and significant time savings in image interpretation, allowing learners more time for practice and understanding [33].

AI can also be used to create personalised-learning paths that are tailored to the learner’s individual progress and needs. Adaptive learning systems analyse learner performance and adjust learning content and methods accordingly. These systems can analyse learners’ performance on different tasks and identify areas for improvement. Based on this performance analysis, AI systems can create customised practice programmes that focus on learners’ specific weaknesses. This significantly boosts the efficiency of the learning process and increases learner motivation and engagement as they receive exactly the support they need.

Another key component of effective training is real-time feedback. AI-based tutoring systems can provide real-time feedback during ultrasound examinations and point out errors or areas for improvement to learners. These systems can recognise and respond to learners’ voice commands and questions, for example by identifying and explaining the relevant anatomical structure. During the ultrasound examination, the AI system can guide the learner through instructions, which enables immediate support and correction and speeds up the learning process. This increases the flexibility and autonomy of learners, as they can practise outside of class hours and without the immediate presence of an instructor [34].

AI can also be used in the simulation of ultrasound examinations and the creation of virtual patient models. These models provide realistic scenarios that help learners develop their skills in a risk-free environment. Virtual patient models can simulate a variety of clinical scenarios, including normal and pathological findings. These models can be interactive, allowing learners to perform various examinations and make diagnoses. Intelligent ultrasound simulators equipped with AI can adapt realistic scenarios in real time and increase the level of difficulty as learners progress. The advantage of these technologies is that they enable realistic replication of clinical scenarios and provide a safe environment in which mistakes can be made and learnt from without putting patients at risk.

The integration of AI and ML into ultrasound training requires continuous research and development. Current studies and pilot projects show promising results, but there is still much room for improvement and innovation. Studies on the effectiveness of AI-assisted learning have shown that learners who train with AI-based systems learn faster and achieve better results. Long-term studies on the effectiveness of AI-based training methods show that the skills acquired are retained in the long term and can be applied effectively in clinical practice.

Despite the numerous advantages, simulation technologies face a number of challenges. The main challenges include the high cost of purchasing and maintaining the equipment, the need for comprehensive training of instructors and the integration of these technologies into existing training programmes. High-end simulators and VR/AR devices are expensive to purchase, and educational institutions must carefully consider how to integrate these technologies into their budgets. Long-term investments in simulation technologies need to be supported by a detailed cost–benefit analysis.

To maximise the benefits of simulation technologies, trainers must also receive comprehensive training. This includes both the technical handling of the equipment and the ability to give effective feedback and support learners. The integration of new technologies into existing training programmes requires careful planning and implementation. It is important that the new methods are seamlessly combined with traditional approaches to ensure comprehensive and coherent training.

The integration of AI and ML into ultrasound training offers enormous opportunities to improve the quality and efficiency of training. Automated-image analysis, personalised-learning pathways, real-time feedback and virtual patient models are just some of the ways in which AI can revolutionise training. Despite the existing challenges, the future prospects are promising and continued research and development in this area will help to take ultrasound training to a new level. The successful implementation and further development of these innovative technologies will be crucial to revolutionise ultrasound education and increase diagnostic capabilities and patient safety.

## Standardisation and certification

Standardisation and certification in ultrasound training are crucial to ensure high quality and consistency in training and practice. They ensure that professionals performing ultrasound examinations have the necessary knowledge and skills to make safe and effective diagnoses. This section examines the current standards and certification processes, their benefits and challenges, and future prospects for standardisation and certification in obstetric ultrasound education.

Standardisation in ultrasound training means establishing and adhering to uniform training guidelines and procedures that ensure that all learners receive comparable and high-quality training. These standards cover various aspects of training, including curricula, teaching methods, practical training and assessment of learners [35].

A key benefit of standardisation is ensuring consistency and quality. Standardised training programmes ensure that all learners acquire the same basic knowledge and skills, leading to greater consistency and quality in patient care. In addition, standardisation enables the comparison of training outcomes between different institutions and regions, making it easier to identify best practises and areas for improvement. Adherence to uniform standards also reduces the risk of misdiagnosis and treatment errors, which increases patient safety.

Certifications are formal confirmations that a professional has acquired the necessary knowledge and skills to perform ultrasound examinations in obstetrics. These certifications are often issued by recognised professional societies and associations and require passing specific examinations.

One example of a certification programme is the multi-stage programme of the German Society for Ultrasound in Medicine (DEGUM) [36, 37]. It comprises three qualification levels, ranging from basic knowledge to specialised skills. Each level requires the passing of theoretical and practical examinations as well as proof of a certain number of examinations performed. Similarly, the AIUM offers certification programmes for various ultrasound applications, including obstetrics. These programmes include both written examinations and practical assessments to ensure that candidates have comprehensive knowledge and practical skills. In the UK, the Royal College of Obstetricians and Gynaecologists (RCOG) offers certification and diplomas in specialised ultrasound techniques in obstetrics and gynaecology. These programmes involve intensive training and rigorous examinations supervised by experienced professionals.

Certifications provide formal proof of competence and ensure that professionals have the necessary skills to perform ultrasound examinations safely and effectively. Certified professionals often enjoy higher professional recognition and have better career prospects. Certifications can also be a prerequisite for certain positions or promotions. Many certification programs also require regular recertification to ensure that professionals continue their education and maintain their knowledge.

Despite the many benefits, standardisation and certification in ultrasound training face some challenges. One of the biggest challenges is the global variability of standards and certification requirements, which can lead to differences in training quality and clinical practice.

Different standards and certification requirements apply in different countries and regions, which makes it difficult to compare and recognise qualifications. In some regions, especially in developing countries, there is a lack of the necessary resources and infrastructure to implement comprehensive standardisation and certification programmes. In addition, participation in certification programmes can be time-consuming and costly, which is a barrier for many professionals.

## Conclusion

**Fig. 2:**
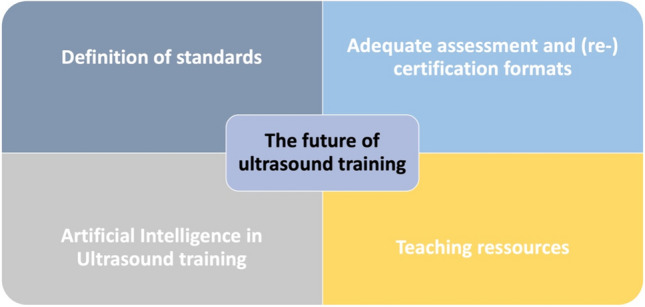
Main focus of innovative approaches for future ultrasound

Ultrasound training in obstetrics is at a turning point where innovative technologies can complement and enhance traditional teaching methods. Simulation technologies, VR/AR, e-learning platforms, gamification and AI offer numerous opportunities to revolutionise medical education and improve the quality of prenatal care. It is crucial that these innovations are carefully implemented and continuously developed to meet the growing demands of modern obstetrics (Fig. 2).

The future of ultrasound training will depend on the successful integration of these technologies, always prioritising the needs of learners and patient safety.

## Data Availability

Not applicable.
